# Warthin’s Tumor of the Parotid Gland With Degeneration to Diffuse Large B-cell Lymphoma: A Case Report and Review of Literature

**DOI:** 10.7759/cureus.36802

**Published:** 2023-03-28

**Authors:** Mauricio Gutierrez-Alvarez, Cynthia Martinez, Ana Priscila Campollo Lopez, Kevin Fuentes, Jorge Alberto Robles Aviña

**Affiliations:** 1 General Surgery, Medica Sur, Mexico, MEX; 2 Plastic and Reconstructive Surgery, Hospital Central Sur de Alta Especialidad Pemex Picacho, Mexico, MEX; 3 Surgical Oncology, Medica Sur, Mexico, MEX; 4 Surgical Oncology, Hospital General Dr. Manuel Gea González, Mexico, MEX

**Keywords:** diffuse large b-cell lymphoma, case report, parotid gland, lymphoma, warthin's tumor

## Abstract

Parotid gland neoplasms are rare; some benign lesions, such as Warthin's tumor (WT), can present as malignant degeneration to carcinomas or, even rarer, to lymphomas. In the literature, there are fewer than 30 reported cases of primary lymphoma of the parotid gland. We present a case of a 65-year-old male patient with a first diagnosis of WT of the parotid gland who later presented a tumor recurrence and underwent a second surgery, reporting diffuse large B-cell lymphoma of the parotid gland. He underwent a right parotidectomy and chemotherapy, and at his 5-month follow-up, he remains free of recurrence. These tumors may look clinically like benign tumors. However, it is essential to be always alert to detect potentially malignant neoplasms and to emphasize examining the lymphoid component of WT to have an early-stage diagnosis of possible lymphomas and treat them before morbidity and mortality increase.

## Introduction

Most parotid gland tumors are benign, the most common of which is a pleomorphic adenoma, followed by Warthin's tumor (WT). Malignant tumors are sporadic; the most frequent are mucoepidermoid carcinoma, adenoid cystic carcinoma, and squamous cell carcinoma [1]. Primary salivary gland lymphomas account for 4.7% to 5% of extranodal lymphomas [2]. Between 70-80% of these malignant salivary gland tumors involve the parotid gland [1,3], followed by the submandibular gland at 18% [1]. Primary lymphomas of the parotid gland account for 1%-4% of parotid tumors [1,4,5]. Degeneration of WT to lymphoma has been documented only as an isolated case report, and there are no more than 30 cases in the literature [6,7]. Clinically, it can be indistinguishable from other gland tumors, which delays diagnosis and worsens the prognosis [3], but with proper management, it has a good prognosis [5].

## Case presentation

A 61-year-old male patient with a history of melanoma in his mother and a paternal grandmother died of unspecified cancer. Positive social ethylism, smoking five cigarettes per day for 30 years. Tonsillectomy at 11 years of age. Onset six months before a first surgical intervention (right parotidectomy) with an increase in volume in the right parotid region of 1 x 1cm with the gradual growth of the tumor. He denies paresthesia or pain, so soft tissue ultrasound is requested, where an increase in volume with distortion of the architecture of the right parotid gland is reported, in addition to ipsilateral lymphadenopathies that would correspond to an inflammatory process, probably chronic. The possibility of concomitant elements of a neoplastic nature is not ruled out, so it is complemented with a tomography of the head and neck reporting a neoproliferative process of the right parotid gland associated with lymphadenopathies in the periphery. The patient was scheduled for a right parotidectomy with modified cervical radical dissection. As a result of these findings, a 6 x 4 x 3 cm tumor was found in the right parotid gland. The histopathology report diagnosed Warthin's tumor and inflamed cervical levels I to V.

**Figure 1:**
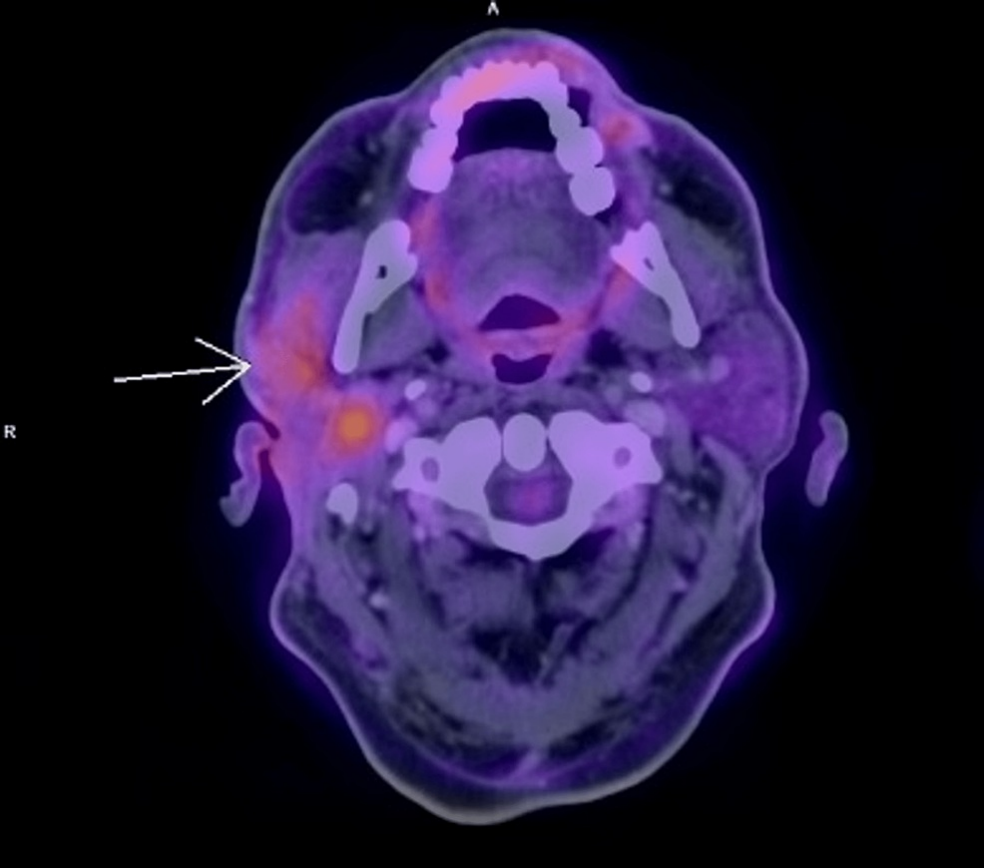
Generalized increase in volume and density of the right parotid gland, compatible with neoformation, indicated with white arrow.

**Figure 2:**
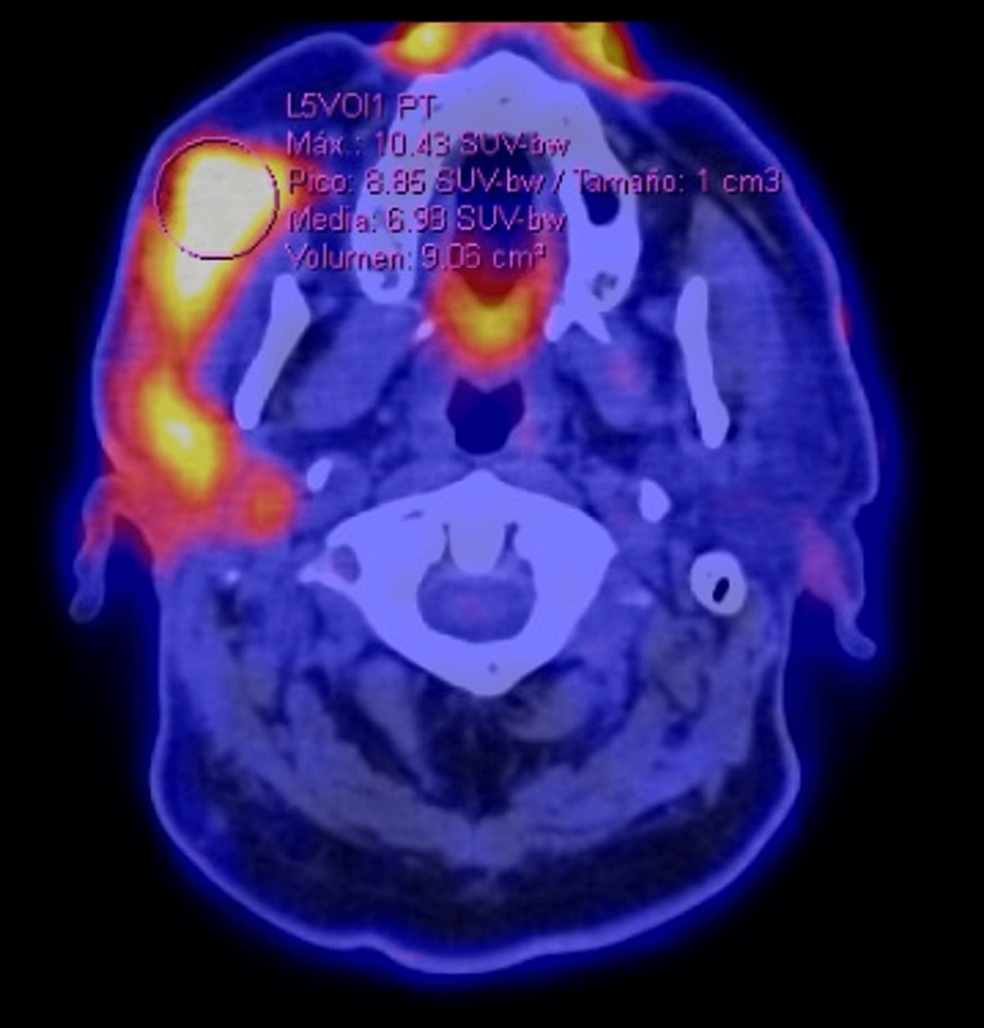
Increased volume and density of the right parotid gland, with increased metabolism and SUVmax up to 10.4, indicated with a circle.

**Figure 3:**
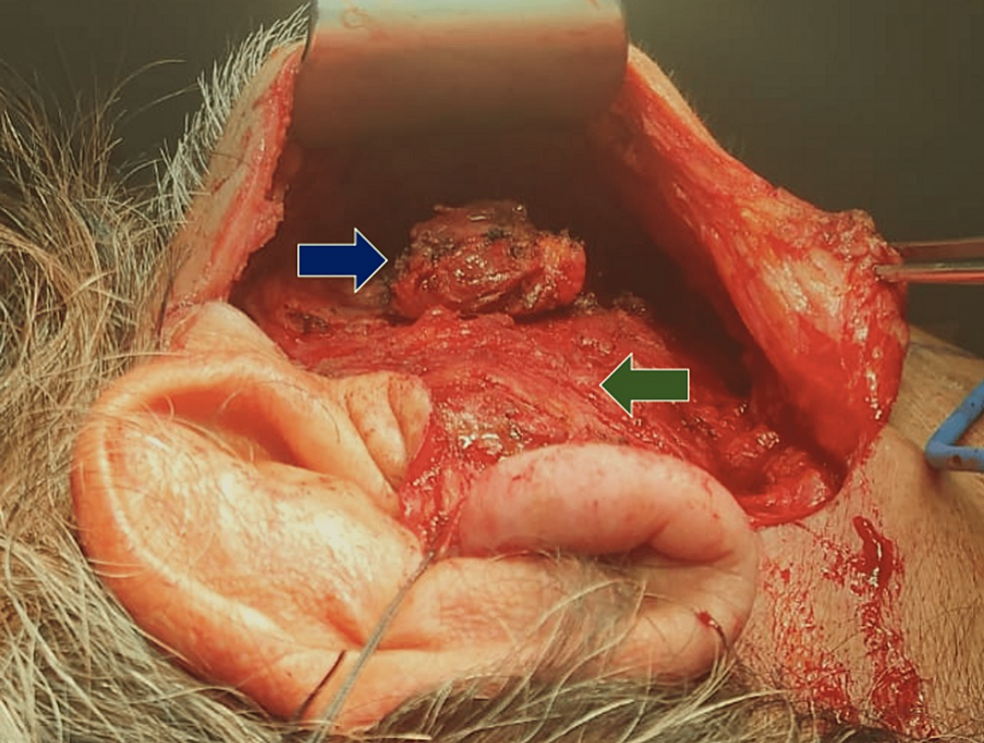
Blair incision for parotid gland approach. With blue arrow, parotid tumor, with a green arrow, masseter muscle.

**Figure 4:**
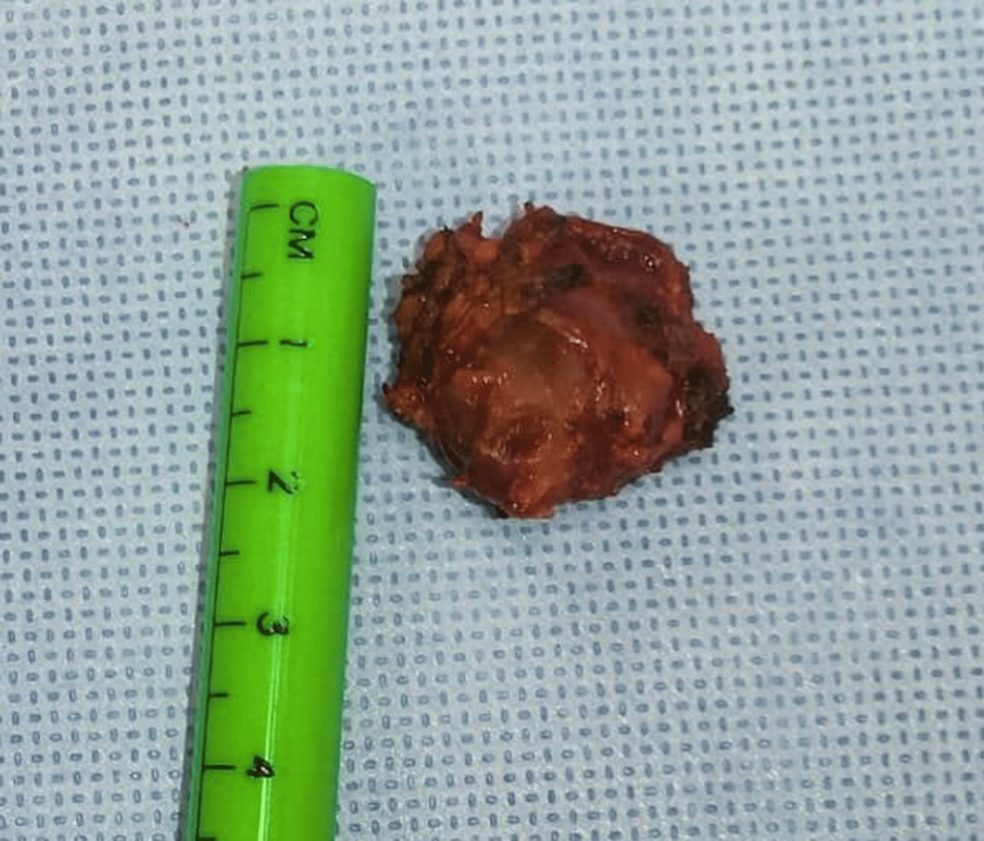
Neoplastic lesion of approximately 2 cm, irregular borders, indurated.

Six months after the surgery, the tumor recurred. A PET-CT with 18 FDG was requested (Figure 1), showing an increase in volume and generalized density of the right parotid gland, suggestive of a neoformation with extension to the ipsilateral masseter muscle. Twenty cycles of prednisone: 20mg for ten days and 10mg for the subsequent ten days, presenting a decrease of 50% of the tumor volume; however, due to the persistence of the symptoms, a new PET-CT with 18 FDG was performed (Figure 2), where a significant increase in metabolism and SUVmax of up to 10.4 was reported (previously 7.7), adding small occipital lymph nodes with increased metabolism, findings suggestive of metabolic progression of the lymphoproliferative disease. Due to these findings, it was staged as Ann Arbor II. It was decided to perform a right malar, parotid, and temporomandibular resection with dissection and preservation of the facial nerve (Figures 3, 4), guided by neuromonitoring.

**Figure 5:**
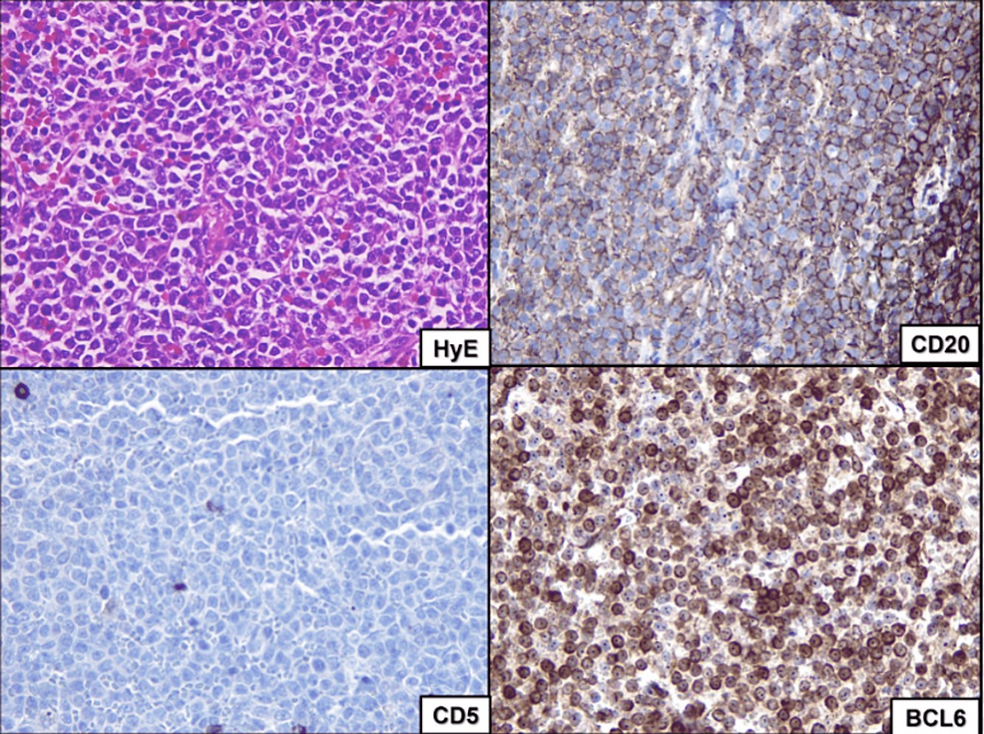
Hematoxylin and eosin (H&E) 40x staining showed a poorly differentiated neoplasm with a solid and diffuse growth pattern consisting of large cells with large nuclei and scanty or even absent cytoplasm. Immunohistochemical study 40x, where the positive marking to CD20 and the negative marking to CD5, shows that the tumor population is of the B lineage, the BCL6 marker was positive, and Cyclin-D1 was negative.

The patient was discharged 2 days after the procedure with a final pathology report of an irregular fragment of 3.8 x 2.5 x 0.9 cm, brown-white with an irregular border, CD20 positive, CD5 negative, BCL6 positive, Cyclin D1 negative, Ki67 positive in 60%, diffuse large cell lymphoma with B immunophenotype (Figure 5). During the postoperative period and follow-up at 5 months, the patient received 4 cycles of R-CHOP as chemotherapy and remained stable without recurrence and asymptomatic.

Hematoxylin and eosin (H&E) staining showed a poorly differentiated neoplasm with a solid and diffuse growth pattern consisting of large cells with large nuclei and scanty or absent cytoplasm. The immunohistochemical study, where the positive marking to CD20 and the negative marking to CD5, shows that the tumor population is of the B lineage, the BCL6 marker was positive, and Cyclin-D1 was negative.

## Discussion

Primary parotid gland lymphomas are rare and, as described in the literature, predominate in elderly patients with a mean age of 65 years [6,7], in the male sex [5-7], and mainly affect the parotid gland [6,7], very similar to the case of our patient.

The most common clinical features are unilateral painless tumors [1,3], as in our case, or bilateral, which occur in 4%-20% of cases [3]. Some other clinical features that may be present are cervical lymphadenopathy, pain, or facial nerve paresis [5]. This nonspecific presentation can make diagnosis difficult and delayed [3]. Differential diagnoses are lymphoepithelial sialadenitis (LESA) and Warthin's tumor (WT) [8]. One percent of WT can be malignant, usually transforming into carcinoma. However, transformation to lymphomas, as in our case, is very rare [7,9], with no more than 30 cases reported in the literature [6,7]. The risk factors for malignancy are not entirely clear; exposure to radiotherapy has been proposed [10] in our case, there was no such history; however, in the cases reported by Ozkök G et al., Li Jun et al., Wang CS et al. and some other authors, they report a history of heavy smoking [6,7,9] which could be a predisposing factor for transformation to lymphoma as in our case. Some diseases, such as Sjogren's syndrome or Mikulicz's disease, are also risk factors for malignant transformation [1,3,10].

Lymphomas are a group of malignant diseases characterized by neoplastic proliferation of the lymphoreticular portion of the reticuloendothelial system [2]. Histologically, most of these neoplasms are of the non-Hodgkin type B group [8]. Within this group, the most frequent subtypes are extranodal marginal zone mucosa-associated lymphoid tissue (MALT) B-cell lymphoma, follicular lymphoma, and diffuse large B-cell lymphoma [1,4,7]. In our case, the most common is diffuse B-cell lymphoma [11]. A characteristic of these neoplasms is that they all originate from B lymphocytes, and cervical lymph node involvement may be associated. This parotid lymphoma originates in intraparotid lymph nodes, parenchymal lymph nodes, or both [8].

As part of the diagnostic approach, computed tomography has been preferred as the initial method because it is easily accessible and provides the location, size, and structures involved in the tumor. Some of the most common CT findings are an ill-defined tumor, a diffuse mixed solid cystic tumor with an ill-defined margin, and multiple solid masses with well-defined borders [1]. However, the findings remain nonspecific [11]. Another study that can be performed and even helps stratify the disease is PET-CT with 18F-labeled fluorodeoxyglucose (18-FDG) [1,5]. Another advantage is its usefulness in disease follow-up [5]. The definitive diagnosis is made through histopathological study, which can be obtained through fine needle aspiration biopsy (FNAB) and core and excisional biopsy. It has been reported that FNAB has a high false negative rate, especially for Warthin's tumor, pleomorphic adenoma, and lymphoepithelial lesions [1], and a sensitivity of 12% [11]; because of this, larger samples are preferred. Even excisional biopsy [1], despite being more invasive, is preferred, with a sensitivity and specificity of 98% and 99%, respectively [11]. The involvement of the salivary gland as the first clinical manifestation of the disease, the histological evidence that the lymphoma affects the parenchyma of the salivary glands, and the architectural and cytological confirmation of malignant character make suspect that this is a primary neoplasm of the parotid gland [3,8] as in the case of our patient, where the first manifestation was the increase in the volume of the parotid gland and the histopathological confirmation was similar to the previously mentioned criteria.

This neoplasm is sensitive to chemotherapy and radiotherapy, so a timely diagnosis has a better prognosis even for function since surgical management can injure nerve structures due to the infiltrative process of the lesion [8]. Some studies report that surgery patients have 35% lower mortality than those who do not undergo parotidectomy [1]. Like other authors such as Duque et al. or Zieliński M et al., we recommend using facial nerve neuromonitoring to improve functional outcomes and avoid unwanted injuries. This follow-up does not affect surgery or its duration but helps to reduce the prevalence of transient facial nerve paralysis after parotid tumor surgery or, even worse, permanent lesions [12,13].

In the study by Gupita A et al., a better survival rate is demonstrated when combined chemotherapy and radiotherapy are combined as treatment than those who only receive chemotherapy or no adjuvant treatment. The currently preferred chemotherapy management is with rituximab, cyclophosphamide, doxorubicin, vincristine, and prednisone (R-CHOP) [11], the same scheme that we have used. In short-term follow-up, we obtained good results without evidence of recurrence.

The prognosis depends on tumor histology, stage, and associated risk factors; low-grade tumors are generally MALT and follicular, and high-grade lymphomas are diffuse large B-cell lymphoma [5], as in our case. The 5-year survival rates for non-Hodgkin's lymphomas are 75% to 83% [5,11] in local diseases and drop to 64% in distant diseases. Despite this, only 16% of patients present at late stages, such as III or IV. Finally, patients with submandibular gland involvement have a mortality rate of 0.6 times lower than when the parotid gland is involved [11].

## Conclusions

Parotid lymphoma is a rare neoplasm that, due to its clinical characteristics, can be confused with more common tumors such as Warthin's tumor (WT) or pleomorphic adenoma; thus, it can go unnoticed and be detected in more advanced stages with a worse prognosis. It is important to be always alert to tumors in the parotid region and maintain a high suspicion to detect potentially malignant neoplasms. On the other hand, we must intentionally review the lymphoid components of the WT because they can degenerate into lymphomas, which will require more specific management associated with surgery and chemotherapy.
